# Contributions of Microdialysis to New Alternative Therapeutics for Hepatic Encephalopathy

**DOI:** 10.3390/ijms140816184

**Published:** 2013-08-05

**Authors:** Liliana Rivera-Espinosa, Esaú Floriano-Sánchez, José Pedraza-Chaverrí, Elvia Coballase-Urrutia, Aristides Sampieri, Daniel Ortega-Cuellar, Noemí Cárdenas-Rodríguez, Liliana Carmona-Aparicio

**Affiliations:** 1 Laboratory of Pharmacology, National Institute of Pediatrics, Mexico D.F. 04530, Mexico; E-Mail: lili_rives@yahoo.com; 2 Section of Research and Graduate Studies, IPN, Mexico D.F. 11340, Mexico; E-Mail: floriano_esa@yahoo.com; 3 Military School of Graduate of Health, Multidisciplinary Research Laboratory, SEDENA, Mexico D.F 11200, Mexico; 4 Department of Biology, Faculty of Chemistry, University City, UNAM, Mexico D.F. 04150, Mexico; E-Mail: pedraza@unam.mx; 5 Laboratory of Neurochemistry, National Institute of Pediatrics, Mexico D.F. 04530, Mexico; E-Mail: elcoballase@yahoo.com.mx; 6 Laboratory of Molecular Biology and Genomic, Faculty of Sciences, University City, UNAM, Mexico D.F. 04150, Mexico; E-Mail: aris_sampieri@yahoo.com.mx; 7 Nutrition Laboratory, National Institute of Pediatrics, Mexico D.F. 04530, Mexico; E-Mail: dortega@unam.mx

**Keywords:** microdialysis, hepatic encephalopathy, alternative therapeutics

## Abstract

Hepatic encephalopathy (HE) is a common complication of cirrhosis, of largely reversible impairment of brain function occurring in patients with acute or chronic liver failure or when the liver is bypassed by portosystemic shunts. The mechanisms causing this brain dysfunction are still largely unclear. The need to avoid complications caused by late diagnosis has attracted interest to understand the mechanisms underlying neuronal damage in order to find markers that will allow timely diagnosis and to propose new therapeutic alternatives to improve the care of patients. One of the experimental approaches to study HE is microdialysis; this technique allows evaluation of different chemical substances in several organs through the recollection of samples in specific places by semi-permeable membranes. In this review we will discuss the contributions of microdialysis in the understanding of the physiological alterations in human hepatic encephalopathy and experimental models and the studies to find novel alternative therapies for this disease.

## 1. Introduction

Encephalopathies are a group of diseases affecting the brain that are manifested through a wide gamut of etiologies and symptomatology. The main causes of encephalopathy are infections, liver damage, anoxia, hypoxia, ischemia and kidney failure.

Hepatic encephalopathy (portosystemic encephalopathy; HE) is a common complication of cirrhosis, of largely reversible impairment of brain function occurring in patients with acute or chronic liver failure or when the liver is bypassed by portosystemic shunts. This leads to a spectrum of neurological impairments, characterized by a myriad of manifestations, diverse underlying liver disorders and a variety of precipitating factors [1], ranging from subclinical brain dysfunction to coma. In the presence of chronic liver disease, HE is a sign of decompensation, while in fulminant liver failure represents a worrying sign and usually indicates that transplantation is required [2]. The mechanisms causing this brain dysfunction are still largely unclear [3,4].

HE is diagnosed when altered mental state accompanies another primary diagnosis such as chronic liver disease, anoxia, or other diagnoses. To avoid complications of late diagnosis, there is an interest to clarify the mechanisms underlying neuronal damage in order to identify markers that will enable early diagnosis, and also to find therapeutic alternatives for improved patient care. The technique of microdialysis consists of sample recollection using semi-permeable membranes in order to evaluate chemical substances (endogenous neurotransmitters or exogenous such as drugs), in extracellular spaces from different tissues. This is not only used to determine the neurochemical abnormalities induced by the encephalopathies, but also to determine the therapeutic effect of various drugs in the treatment of this pathologic condition. Therefore, in this review we will discuss the contributions of microdialysis in the understanding of the physiological alterations that underlie hepatic encephalopathy in the human and experimental models and studies to find alternative therapies for this disease.

## 2. Biochemistry Alterations in HE

HE is a series of pathological conditions affecting the brain, which is composed of the intracranial components of the central nervous system (CNS). Symptoms of HE can include confusion, disorientation and poor coordination. HE is caused by complication of both acute and chronic liver disease. It is well accepted that high ammonia levels are a dominant explanation of the pathogenesis of HE. Briefly, ammonia is a byproduct of the metabolism of nitrogen-containing compounds, and is most commonly eliminated through the formation of urea in the liver. However systemic accumulation of ammonia [5] due to impaired liver function or a portosystemic shunt, compromises this function and the extrahepatic metabolization of ammonia by another tissues, such as brain and muscle becomes more important [6]. However, it is noteworthy, that the pathophysiology of chronic HE is apparently multifactorial; evidence has emerged for roles of other concurrent factors (neurotoxins, hyponatremia and inflammation).

### 2.1. Etiology

The liver has a central detoxifying role in the body due to its ability to neutralize many toxic chemicals absorbed from the gastrointestinal tract as well as others produced as byproducts of normal metabolism. Most of these toxins reach the liver through the portal venous system and progressing through the low flow hepatic sinusoids; these substances are effectively captured and detoxified by hepatocytes. Hyperammonemia (ammonia blood levels higher than 50 μmol/L) is the key biomarker of HE. The ammonia elevation is mainly caused by the inability of the liver to transform ammonia to urea via the urea cycle in periportal hepatocytes, the diminished glutamine synthesis in centrilobular hepatocytes and the portosystemic shunts. The pathophysiology in most cases consists of several parallel mechanisms that must be considered. For instance, constipation may cause hyperammonemia due to delayed transit rate and subsequently increased absorption of ammonia, but the local pH and the composition of the colonic flora can effectively modulate absorption. It has been long supposed that the urease-producing colonic bacteria break down proteins, urea and possibly amino acids to ammonia, which is then absorbed into the portal circulation [7,8]. This results in pooling of various toxins into the systemic circulation and eventually reaching the brain and other organs. In addition to these hemodynamic changes, the effective hepatocyte mass is significantly reduced in cirrhosis, thus it can be easily overwhelmed by relatively small amounts of toxins [9]. Normal brain function requires anatomical brain integrity, sufficient energy production, and efficient synapse neurotransmission, all of which are impaired in HE.

The ammonia accumulation alone does not single-handedly explain all the neurological disorders of HE. Shawcross *et al.* proposed that inflammatory response is another condition that contributes to HE. Patients with HE have elevated levels of inflammatory markers in serum such as C-reactive protein, and interleukin (IL)-6 [10]. Although the mechanism of this impairment is not very clear, several factors and pathways interact together, resulting in the CNS dysfunction, which is manifested clinically as varying degrees of HE [11].

### 2.2. Clinical Characteristics

Patients with HE display a variety of neuropsychiatric abnormalities. The clinical features and presentation of HE vary based on its severity, ranging from disturbances affecting life quality, abnormal sleep patterns, lack of awareness, increased reaction times, impairments in cognitive and mental function, behavior and personality alteration, and disturbances in attention and coordination to transient neurological symptoms (asterixis or flapping tremor, combined with characteristic electroencephalographic abnormalities) [12,13]. Disturbance in the diurnal sleep pattern is a common early manifestation of HE and is related to altered melatonin secretion. More advanced neurologic features of HE include bradykinesia, asterixis, hyperreflexia, and transient decerebrate posturing, with advanced disease, cerebral edema develops secondary to astrocyte swelling and leads to altered states of consciousness, varying degrees of confusion, stupor, coma and death [1,14]. The HE symptoms are generally reversible, suggesting a metabolic etiology, therefore prevention and effective treatment of HE may have important prognostic implications in patients with chronic and acute liver failure.

### 2.3. Therapeutic Approach

Treatment of encephalopathy varies with the primary cause of the symptoms; consequently, not all cases of encephalopathy are treated in the same way. Most treatment approaches derive from experience with episodic portosystemic encephalopathy. Since deterioration of cognitive function in patients with liver cirrhosis is primarily triggered by precipitating factors [15], consistently avoiding these factors is also paramount for patients with HE. Precipitating factors for development of episodic HE are as follows: gastrointestinal bleeding, hyperkalemia/hyponatremia, constipation, sedatives and tranquilizers, electrolyte imbalances, infections, trauma, dehydration and uremia. Treatment of HE consists of the following goals: (i) lowering blood and cerebral ammonia levels, (ii) dealing with precipitating factors of hyperammonemia and accumulation of toxic metabolites, and (iii) dealing with the consequences of hyperammonemia and accumulation of toxic metabolites. Liver transplantation is indicated for patients with fulminant or subfulminant liver failure associated with HE and is known to significantly improve HE in patients with cirrhosis [16].

#### 2.3.1. Dealing with Precipitating Factors of Hyperammonemia and Accumulation of Toxic Metabolites

In addition to targeting the precipitating factor, several therapeutic methods have been used to reduce the ammonia load. Synthetic disaccharides are widely used in the treatment of HE despite the lack of strong scientific evidence demonstrating their efficacy [17–19]. Studies targeting this process have shown mild effect of Ornithine-Aspartate and sodium benzoate in lowering serum ammonia levels and improving HE [20,21].

#### 2.3.2. Lowering Blood and Cerebral Ammonia Levels

It has been demonstrated that dietary protein restriction in cirrhotic patients does not ameliorate or reverse the course of HE [22]. As a result, a daily protein intake of 1–1.6 g/kg of body weight can be safely administered to a patient with HE, as a positive nitrogen balance is necessary to promote liver regeneration and increased capacity of skeletal muscles to remove ammonia in the form of glutamine [23]. In patients with refractory HE, vegetable protein based diet may be recommended [24,25].

#### 2.3.3. Pharmacological Approach

The benzodiazepine receptor antagonist flumazenil has shown some success in reversing HE, yet its effect was short-lived. Studies have indicated that the gamma amino-butyric acid (GABA)-receptor complex may contribute to neuronal inhibition in HE [24,26].

#### 2.3.4. Novel Approaches and Strategies under Development

Genetically engineered bacteria with the ability to metabolize ammonia at an increased rate have been used in experimental animal models in order to reduce its ammonia levels [27]. The spherical adsorptive carbon AST-120, was able to reduce ammonia levels in experimental animal models [28]. HPN-100 is a pro-drug which, although not yet tested on patients with liver disease, is thought to ameliorate the clinical condition of HE. Acarbose (C_25_H_43_NO_18_) is an alpha-glucosidase intestine inhibitor, affecting metabolism of dietary nitrogen and thereby reducing production of ammonia [29]. Sildenafil has been used in order to improve the cerebral function of patients with HE by increasing the levels of extracellular cGMP [30]. According to current evidence, inflammatory mediators also play an important role in alterations observed in patients with HE (36), and based on this observation in animal models, the ability of indomethacin and ibuprofen to ameliorate the cerebral symptoms in HE patients has been proven [31,32].

### 2.4. Prognosis

The prognosis for a patient with encephalopathy depends on the initial causes and, in general, the length of time it takes to reverse, stop, or inhibit those causes. Consequently, the prognosis varies from patient to patient and ranges from a complete recovery to a poor prognosis that often leads to permanent brain damage or death.

Despite the impressive advances in our understanding of the several pathophysiological mechanisms involved in HE, treatment options remain an unmet clinical need, accompanied by considerably high mortality rates. Studies have indicated that HE affects 30% to 45% of patients with cirrhosis and a higher percentage may be affected by a minimal degree of encephalopathy [30,33]. This subclinical or minimal HE affects up to 80% of patients with cirrhosis [31]. Patient prognosis is very poor; probability of five-year survival is 16% to 22%, compared with that of 55% to 70% in cirrhotic patients without HE [32,34,35]. Five years after diagnosis of cirrhosis, there is a 26% probability for development of at least one episode of HE [36]. It is important to recognize that HE is mostly reversible, and that rather than a worsening of hepatocellular function, identification of a precipitating cause and successful treatment leads to resolution of HE in more than 80% of patients [9,11]. Liver transplantation is the ultimate treatment for curing liver disease, obliterating HE. Before the availability of liver transplantation, acute liver failure was associated with 80% to 90% mortality, especially in patients who progressed to grade 3 or 4 of HE [37]. However, with successful liver transplantation more than 90% of patients survive one year and 75% survive five years post-transplant.

## 3. Microdialysis Technique

### General Characteristics

Microdialysis was proposed to continuously collect samples in specific and different areas; this procedure allows the real time study of the chemical exchange between cells that occur in extracellular space, in contrast to other sampling schemes such as blood sampling that does not reflect specific changes. It also provides information without the need to dissect tissue samples to gather information that would be a static picture of biochemical events [38]. This experimental procedure consists of a closed system, in which a thin tube with a dialysis membrane is inserted into a particular region (liver, skin, brain and others tissues) where the cannula can be fixed. The principle of microdialysis is based on the physicochemical phenomenon of passive diffusion [39–44]; this is a spontaneous and irreversible process in which a substance in solution is transported as a result of movement of its particles. This movement is constant and random, molecules can move in one direction or the other interchangeably. However, if one takes into account the number of particles of a given substance is higher in places that are in high concentrations, the predominant shift to low concentration sites, therefore establishing a net diffusion of the substance from the sites of high to low concentration sites. It is worth mentioning that the semi-permeable membranes are characterized by pore size, so only the low molecular weight molecules are able to go through this barrier [41,44–48].

**Figure 1:**
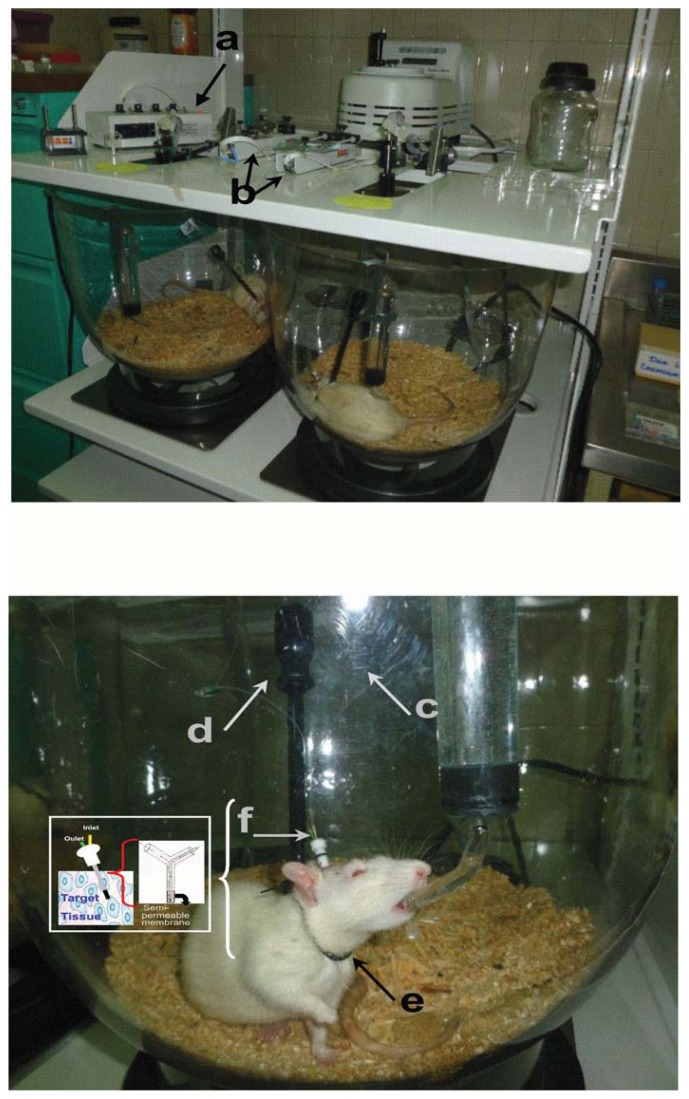
Representative images of microdialysis system for experimental models *in vivo*. The system consists of velocity controller (**a**) through which the vehicle is pushed; pumps (**b**) used for continuous infusion of analyte to be tested; the microdialysis cannula having an inlet tube (**c**); outlet tube (**d**) for recovery of the substance of interest; as well as clamping collar that is placed on experimental animals to have free movement (**e**); microdialysis cannule (**f**). Microdialysis system from Neurochemistry Laboratory of the Pediatric National Institute.

Microdialysis involves the transport of a vehicle (perfusion) by an inlet tube at a continuous and defined flow through a semipermeable membrane which is implanted in the tissue of interest (brain, heart, tumor, liver and skin) and recollected by an outlet tube for posterior analyte quantification, in the case of the *in vivo* experimental models (Figure 1), as well as in humans, and in a solution of known concentration in the case of *in vitro* experiments. Aqueous solutions are commonly used with concentrations of sodium and potassium ions in low concentrations, without the presence of proteins or at very low concentrations that resemble the extracellular space to study. In some cases, the vehicle may contain proteins to prevent drug sticking to the sides of the cannula [46,47,49–51].

Several factors affect this diffusion process in the microdialysis semipermeable membrane, and as such, the recovery of the substances of interest, these are: (a) the flow rate, where recovery is inversely proportional to the infusion rate and the recovery of the substance of interest is close to one hundred percent when the flow is near zero and is minimum when it is fast; (b) the membrane’s composition, there are over 30 different materials to produce membranes which can be directly derived from natural products, semi-synthetic and fully synthetic; (c) the presence of surface charges that reduce the recovery of certain molecules also charged, which does not happen with neutral molecules; (d) total membrane area (length), where recovery is directly proportional to the total area; and (e) temperature of the system, 37 °C facilitates the diffusion process.

Recently, this methodology has many applications in several research areas, due to its use to monitor the release of substances in different tissues in conscious and anesthetized animals. It has also been used in surgical procedures, in monitoring patients in intensive care with traumatic brain injury and subarachnoid hemorrhage, as well as in the research of diseases such as epilepsy [52,53], Alzheimer’s disease [54,55], Parkinson’s disease [56,57] and encephalopathies [58–60].

## 4. Microdialysis in the Study of Encephalopathies

Encephalopathies are a group of diseases that endanger the patient’s life, so the interest is not only to study pathophysiological mechanisms, but also potential therapeutic agents. A wide range of experimental procedures has been used for this purpose, including microdialysis. The first study applying this procedure was performed in the early nineties by Yokel *et al.* [58], where microdialysis was used to determinate aluminum in blood, liver and brain. Microdialysis has been used to study biochemical changes in posthypoxic encephalopathy [61], hypoxic-ischemic encephalopathy [62–64], encephalopathy associated with septic shock [65], thiamine deficiency encephalopathy [66–69], viral encephalopathy [70] and hepatic encephalopathy described in more detail in the next section of this review.

### 4.1. Microdialysis Contributions in Understanding Mechanisms That Induce HE

The main contributions of the use of microdialysis in the study of the underlying mechanism of HE are described in Table 1.

### 4.2. Microdialysis Applied to the Study of Hepatic Encephalopathy Therapeutic Treatments

#### 4.2.1. l-Ornithine and l-Ornithine-l-Aspartate Study

Vogels *et al*. [94] demonstrated that the l-ornithine (ORN) and l-ornithine-l-aspartate (OA) therapeutic treatment had beneficial effects on the symptomatology of rats with hyperammonemia-induced encephalopathy by portacaval shunt (PCS). They found that ORN and OA treatment, decreases ammonia concentration in blood (34% and 39%) and in brain (42% and 22%), increases urea production (39% and 86%) and significantly increases glutamine and lactate concentrations in brain. However the effect of ornithine should be taken with care since this substance induces high brain extracellular levels of glutamate and aspartate, suggesting a possible overstimulation of *N*-methyl-d-aspartate (NMDA) receptors.

#### 4.2.2. Venlafaxine Studies

Studies of patients with chronic HE, presenting affective symptoms and antidepressant drug treatment, find monoaminergic perturbations together with affective symptoms. Venlafaxine (VEN) is an antidepressant, serotonin-norepinephrine reuptake inhibitor that is used in the treatment of patients with HE. The liver impairment present in HE patients may induce pharmacokinetic alterations of the antidepressant drug, which in turn can modify monoaminergic function. Wikell *et al*. [95,96] have studied these possible alterations; in fact they have determined that rats with PCS, VEN (10 mg/kg) administered in a single dose (subcutaneous) and daily during 14 days (continuous delivery by osmotic minipumps), exhibit both pharmacokinetic and pharmacodynamic alterations.

VEN administered as a single subcutaneous challenge (5 mg/kg) to PCS rats resulted in elevated levels of VEN in serum, brain parenchyma, and brain dialysate about 300 min after the injection. Therefore, this result suggests that when the dose of VEN administered to experimental HE was reduced by 50%, important pharmacokinetic alterations are present in animals with this condition [97], similar to the studies using the dose of 10 mg/kg [96].

#### 4.2.3. Citalopram Studies

Citalopram (CIT) is an antidepressant drug of the selective serotonin reuptake inhibitor used in patients with HE that displays neuropsychiatric symptoms such as affective disturbances. The simultaneous pharmacokinetic and pharmacodynamic outcome of the commonly used serotonin-selective thymoleptic drugs in liver-impaired subjects with HE is not totally understood at present.

Berqvist *et al*. [98] studied the effects of neocortical administration of CIT (1.0 microM), and CIT (5 mg/kg subcutaneously) on brain 5-hydroxytryptamine (5-HT) release in PCS in experimental chronic HE. They found that neocortical administration of CIT increased the brain 5-HT output in the same way in PCS as in sham-operated rats; these data do not explain the increased 5-HT turnover and unchanged release in PCS rats by an accelerated brain 5-HT reuptake. They administered CIT (5 mg/kg subcutaneously) and this resulted in a pronounced decrease of brain 5-HT release in PCS rats in comparison to the sham-operated controls. This may be due to a higher susceptibility to indirect activation of the 5-HT1A autoreceptor in experimental portal-systemic encephalopathy. Their experiments with KCl (60 mM) challenging the presence of locally CIT (1 microM) induced an increase of 5-HT response higher in PCS rats than in sham-operated rats and confirmed an abnormal increase of 5-HT available for depolarization-induced release in PCS rats. These results suggested that CNS 5-HT-active drugs perhaps pose a potential hazard in patients with liver failure with or without HE.

In addition, Apelqvist *et al*. [99] investigated the effects of chronic treatment with CIT (10 mg/Kg day) in the frontal neocortex of rats with and without PCS in the 5-HT, 5-HIAA, noradrenaline (NA), and dopamine (DA) output. The rats with PCS had a higher increase in CIT levels (2–3-fold) than rats undergoing a sham treatment with CIT in all compartments. This treatment induced neocortical output differences between PCS rats and control rats within 5-HT and DA systems but not in the NA system. Their data suggest pharmacokinetic and pharmacodynamic changes at doses equal to chronic treatment with CIT in PCS rats, changes that were not observed in sham rats. This author stated that although there are pharmacokinetic and pharmacodynamic alterations with CIT treatment in PCS rats, the beneficial behavioral response remains.

#### 4.2.4. Lubeluzole Study

Lubeluzole is a neuroprotectant effective in the treatment of experimental stroke in rats, mainly by inhibition of the glutamate-activated nitric oxide pathway and also by counteracting osmotic stress (a mechanism associated with the release of the active amino acid taurine). Zielinska *et al*. [100] showed that lubeluzole, administered i.p., decreased the high (50 mM) K^+^-evoked accumulation of taurine in striatal microdialysates of healthy rats by 25% and by 34% in rats with thioacetamide-induced hepatic failure, suggesting increased extracellular taurine in ongoing HE. These data indicate that lubeluzole could be effective in ameliorating ionic or osmotic stress in rats with hepatic failure.

#### 4.2.5. Sildenafil Study

Patients with liver disease with overt or minimal HE display impaired intellectual capacity and the underlying molecular mechanism remains unknown. An interesting fact is that the rats with portacaval anastomosis or with hyperammonemia without liver failure also show impaired learning ability and impaired function of the glutamate-nitric oxide-cyclic guanine monophosphate (glutamate-NO-cGMP) pathway in the brain. Erceg *et al*. [26] hypothesized that pharmacological manipulation of this pathway (glutamate-NO-cGMP pathway) could restore learning ability. They showed by *in vivo* brain microdialysis that chronic oral administration of sildenafil (an inhibitor of the phosphodiesterase that degrades cGMP) normalizes the function of the glutamate-NO-cGMP pathway and extracellular cGMP in brain *in vivo* in rats with portacaval anastomosis or with hyperammonemia. They determined that impairment of learning ability in rats with chronic liver failure or with hyperammonemia is the result of impairment of the glutamate-NO-cGMP pathway and that the chronic treatment with sildenafil normalizes the function of the pathway and restores learning ability in rats with PCS or with hyperammonemia.

#### 4.2.6. Ibuprofen Study

Patients with HE show altered motor function, psychomotor slowing, and hypokinesia, which are reproduced in rats with PCS. The neurochemical alterations induced by hypokinesia in PCS rats are attributed to the increase of extracellular glutamate in substantia nigra pars reticulate (SNr), but the mechanisms by which liver failure leads to increased extracellular glutamate in SNr remain unclear. However, it has been seen that inflammation acts synergistically with hyperammonemia to induce neurological alterations in HE and in this way the inflammation alterations may be contributing to motor alterations in HE. For this reason Cauli *et al*. [101] investigated whether treatment with ibuprofen, an anti-inflammatory, is able to normalize extracellular glutamate in SNr and/or to improve hypokinesia in PCS rats. They found that ibuprofen at 15 or 30, (but not at 5) mg/kg day, completely eliminated hypokinesia and restored normal motor activity. This supports the hypothesis that inflammation is the main contributor to the induction of hypokinesia in HE. These data also suggest that therapeutic treatment of inflammation for motor deficits in patients with this pathology could be beneficial.

## 5. Effect of Natural Products in HE

Currently, few studies have demonstrated the effect of natural products in the treatment of HE and other liver failures. Subash and Subramanian [102] demonstrated that morin, a bioflavonoid constituent of many herbs and fruits, significantly improved the status of antioxidants vitamins A, C and E, reduced glutathione, glutathione peroxidase, superoxide dismutase and catalase and decreased the levels of ammonia, urea, lipoperoxidation, hydroperoxides and liver markers enzymes in a murine model of hyperammonaemia induced with ammonium chloride. Kaziulin *et al*. [103] demonstrated that bioactive substances with extracts of plants (*Bacopa monneria*, *Gingko biloba*, Cat’s Claw, Gotu Kola and Rosemary) in the diet of 66 patients with HE showed significant improvement of clinical signs, psychometric tests, electroencephalography and serum biochemistry than in group with standard therapy, during periods of 2 to 5 weeks. Mitra *et al.* [104] showed the protective effect of HD-03 in CCl_4_-induced HE in rats. HD-03 is a multi-herbal formulation consisting of *Solanum nigrum* L, *Cichorium intybus* L, *Picrorrhiza kurroa* Benth, *Tephrosia purpurea* L and *Andrographis paniculata* Nees. This formulation prevented the elevation of serum hepatic transaminases and blood ammonia levels. A histomorphometric evaluation of liver and brain showed a protective effect of the herbal formulation. HD-03 treatment brought about an appreciable reduction in the number of astrocytes and absence of their hyperthophy in the brain and preservation of hepatocellular function. Moreover, Harputluoglu *et al*. [105] showed the protective effect of *Gingko biloba* in induced fulminant hepatic failure in rats by thioacetamide. In this work, serum levels of ammonia, serum and liver lipoperoxidation levels, liver necrosis and inflammation were diminished with the use of *Gingko biloba*. Other studies demonstrated the beneficial effect of probiotics, acetyl-l-carnitine and diets based on vegetable protein for HE treatment [21,106–119]. The data suggest that lactulose and dietary intervention with probiotics enhance intestinal health and influence the gut-liver axis, including modulation of the intestinal microflora, modification of intestinal barrier function, and immunomodulation [111,114,120]. Acetyl-l-carnitine induces the recovery of neuropsychological activities and ureagenesis, decreases the severity of mental and physical fatigue and depression cognitive impairment, enhances the mitochondrial function, improves cerebral energy levels and is also a free radical scavenger [121–124]. Finally it has been shown that a modified diet based in vegetable protein improves mental status, nitrogen balance and plasma amino acids [116].

## 6. Conclusions

The main contributions of microdialysis to the underlying mechanisms involved in the induction of HE and to the study of new alternative therapeutics for this disease have been reviewed. With this technique, it has been possible to reach the following conclusions in HE: the involvement of the glutamatergic system (by measuring glycine levels and by using MK-801 and memantine), of glutamate-NO-cGMP, of amino acids, and of extracellular glutathione (GSH), but not of QUIN and TRP. In addition, it has been found that intracranial pressure may be a sensitive marker, that the excitability of the brain may serve as a key in the onset of HE, and that the dopaminergic system, the noradrenergic system and the serotonergic neurotransmission are altered in HE.

Microdialysis has contributed to the study of new alternative therapeutics. l-ornithine and l-ornithine-l-aspartate, venlafaxine, citalopram, lubeluzole, sildenafil and ibuprofen were considered in this review. This technique has evaluated the following: the effect of ORN and OA on ammonia and metabolites levels, the pharmacokinetic and pharmacodynamics alteration of VEN, the effect of CIT on brain 5-HT release and the normalization of the function of the glutamate-NO-cGMP by sildenafil. However, this technique has not been used to evaluate the therapeutic effect of natural products. In conclusion, microdialysis has played a central role in the study of the mechanisms involved in the induction of HE as well as in the study of new alternative therapeutics for this disease.

## Figures and Tables

**Table 1:** Evidence of biochemical changes-induced by HE.

Encephalopathy	Experimental model	Evidence	Author
HE	Pigs	It has been shown the association between extracellular brain ammonia and intracranial pressure (ICP), suggesting that ICP could serve as marker for HE.	[71]
HE	Rats administered locally with fluoroacetate in the hippocampus by microdialysis	Ammonia alters the function of astrocytes, facilitating its entry into the brain. This physiological fact contributes to the development of HE.	[72]
HE	Portacaval shunt (PCS)	Quinolinic acid (QUIN) and l-tryptophan (l-TRP) are not involved in HE. Elevated l-TRP availability increased the QUIN levels to a similar degree in both sham and PCS rats.	[73]
HE	Rats with ischemic liver failure	The significant three-fold increase of extracellular glycine measured by *in vivo* cerebral microdialysis suggests the participation of NMDA.	[74]
HE	Rats administered with galactosamine	The blockage of NMDA receptors by continuous administration of MK-801 or memantine induces protection against acute liver failure. The blockage of NMDA receptors increases the survival rate from 23% to 62% in rats.	[75]
HE	Rats with hyperammonemia induced by intracerebral ammonia infusion	The marked elevation in glutamate levels suggests that high ammonia levels may increase the excitability of the brain and this condition may serve as a key in the onset of HE.	[76]
HE	Rats with liver failure induced by thioacetamide	Experimental data show a significant increase in extracellular hippocampal glutamate concentration.	[77]
HE	Rats with hypothermia	Beneficial effect of hypothermia in rats with hepatic devascularization that induces ALF is mediated via mechanisms involving reduced blood-brain transfer of ammonia and/or reduction of extracellular brain glutamate concentrations.	[78]
HE	PCS and Sham rats	Participation of glutamate-nitric oxide-cyclic guanosine monophosphate (cGMP) was shown. The basal NOS activity, nitrites and cGMP are increased in cortex of rats with hyperammonemia or liver failure. These are associated to increased inducible NOS expression. It was found; in both animal models and in neurons exposed to ammonia, an impaired NOS activation by NMDA.	[79]
HE	Hyperammonemic rats	It was found increased tonic activation of NMDA receptors leading to reduced activity of nNOS and of the glutamate-NO-cGMP pathway.	[80]
HE	Rats with ALF	Amino acids play a role in the pathogenesis of hepatic encephalopathy in ALF. They found that extracellular concentration of the neuroactive amino acids glutamate, taurine and glycine were increased, whereas extracellular concentration of aspartate and GABA were unaltered and that glutamine of decreased.	[81]
HE	Rats with subclinical hepatic encephalopathy induced by intraperitoneal thioacetamide	In cerebral cortical microdialysates of rats was found that dialysate concentration of the neuroactive amino acids taurine (Tau), glutamate (Glu) and aspartate (Asp) were 30% to 50% higher than that found in control.	[82]
HE	Rats with HE induced by ALF	The precursors of monoamines, as well as monoamines and their metabolites, altered neuronal excitability and contribute to the characteristics of HE extracellular brain concentrations of aromatic amino acids (AAAs) and of valine and leucine (precursors of monoamine neurotransmitters) were elevated 2 to 4-fold following hepatic devascularization and these increases were significantly correlated to arterial ammonia concentration.	[83,84]
HE	Animals administered with flumazenil	Extracellular concentration of 3,4-dihydroxyphenylacetic acid, a metabolite of dopamine, decreased to 39% compared with sham-operated animals, without changes in the dopamine level. The treatment with flumazenil completely abolished the decrease in the metabolite. Although in this study the glutamate level in the injured animals decreased to 42% of that in sham-operated animals, there are not increases in the glutamate levels in animals treated with flumazenil. In conclusion, the restoration of the central dopaminergic function could be a relevant factor in the improvement of HE.	[85]
HE	Hyperammonemics rats	The locomotion induced by injection of the mGluR agonist dihydroxyphenylglycine (DHPG) into nucleus accumbens was increased. Also in control rats DHPG increased extracellular dopamine (400%), but glutamate was unchanged. Whereas that in hyperammonemic rats DHPG increased extracellular glutamate (600%), effect prevent by blocking mGluR1 receptor. This result suggests that modulation of locomotor and neurochemical functions by mGluRs in nucleus accumbens are strongly altered in hyperammonemia.	[86]
HE	Rats with acute HE induced thioacetamide administration	The impairment of modulation of striatal DA discharge and metabolism by glutamate, acting at NMDA receptors, contributes to the motor disturbances in HE.	[87]
HE	Rats with liver failure due to PCS	The activation of the normal neuronal circuit in VP, SNr, MDT, and VMT was determined using in vivo brain microdialysis. It is suggested that DHPG-induced increase in dopamine would activate the normal neuronal circuit, while an increase in glutamate would activate the alternative circuit.	[88]
HE	Model of chronic HE, by acute comainducing by ammonium acetate (5.2 mmol/kg, i.p.)	The serotonergic system is also affected in the HE. The extracellular levels of 5-hydroxytryptamine (5-HT) is unaltered and that of its major metabolite, 5-hydroxyindole-3-acetic acid (5-HIAA), is increased in the frontal neocortical of PCS rats. Results suggest that the increase brain ammonia may increase neuronal 5-HT release in HE, which in turn could be involved in the severe stages of HE.	[89]
HE	Rats with thioacetamide (TAA)-induced HE	Serotonergic neurotransmission is altered in the frontal cortex of rats with thioacetamide (TAA)-induced HE. Where found that 5-HIAA and high K^+^-evoked 5-HT release were increased.	[90]
HE	Rats with acute liver failure	Noradrenergic system is affected and the central noradrenergic mechanisms may contribute to the central nervous system manifestations of HE. They showed that the increase of extracellular brain concentrations of the noradrenaline (NA) from frontal cortex and thalamus is associated to loss of NA transporter sites and depletion of central NA stores.	[91]
HE	Rats where the administration of ammonium chloride (ammonia)	Extracellar glutathione (GSH) is involved in the ammonia toxicity present in HE microdialysis probe to the rat prefrontal cortex increased GSH. This increase is abrogated by fluoroacetate, an inhibitor of astrocytic energy metabolism, and by buthionine sulfoximine, an inhibitor of glutathione synthesis. Their results suggest that in rats with hiperammonemia promote GSH synthesis and this may improve the availability of precursors for GSH synthesis in neurons and their resistance to ammonia toxicity present in HE.	[92]
Fulminant hepatic encephalopathy	Intracerebral microdialysis during cardiac resuscitation in rats	They measured the chemical markers of energy metabolism glucose, lactate, pyruvate, and the marker of cell membrane damage glycerol and found that all markers with exception for subcutaneous glucose, showed a sudden and significant increase during resuscitation and a prolonged period afterwards and finally after some hours all values returned to normal.	[93]
Portal-systemic encephalopathy	PCS rats	They evaluated the participation of serotonin system in PCS, and found an increased brain tissue and extracellular concentrations of serotonin in neocortical region of the rats with this encephalopathy.	[89]
